# The neurotoxic effect of clindamycin - induced gut bacterial imbalance and orally administered propionic acid on DNA damage assessed by the comet assay: protective potency of carnosine and carnitine

**DOI:** 10.1186/1757-4749-5-9

**Published:** 2013-04-12

**Authors:** Afaf El-Ansary, Ghada H Shaker, Amina R El-Gezeery, Laila Al-Ayadhi

**Affiliations:** 1 Department of Biochemistry, College of Science, King Saud University, P.O Box 22452, Riyadh 11495, Saudi Arabia; 2 Department of Physiology, Faculty of Medicine, King Saud University, P.O Box 22452, Riyadh 11495, Saudi Arabia; 3 Autism Research and Treatment Center, Riyadh, Saudi Arabia; 4 Shaik AL-Amodi Autism Research Chair, King Saud University, Riyadh, Saudi Arabia; 5 Department of Microbiology and immunology, College of Pharmacy, Zagazig University, Zagazig, Egypt

**Keywords:** Propionic acid, Clindamycin, Tail length, Tail moment, Carnosine, Carnitine, Autism, Neurotoxicity

## Abstract

**Background:**

Comet assay is a quick method for assessing DNA damage in individual cells. It allows the detection of single and double DNA strand breaks, which represent the direct effect of some damaging agents. This study uses standard comet quantification models to compare the neurotoxic effect of orally administered propionic acid (PA) to that produced as a metabolite of bacterial overgrowth induced by clindamycin. Additionally, the protective effect of carnosine and carnitine as natural dietary supplements is assessed.

**Methods:**

Single cell gel electrophoresis (comet assays) were performed on brain cortex and medulla samples after removal from nine groups of hamsters including: a control (untreated) group; PA-intoxicated group; clindamycin treated group; clindamycin-carnosine group and; clindamycin-carnitine group.

**Results:**

There were significant double strand breaks recorded as tail length, tail moment and % DNA damage in PA and clindamycin-treated groups for the cortex and medulla compared to the control group. Neuroprotective effects of carnosine and carnitine were observed. Receiver Operating Characteristics curve (ROC) analysis showed satisfactory values of sensitivity and specificity of the comet assay parameters.

**Conclusion:**

Percentage DNA damage, tail length, and tail moment are adequate biomarkers of PA neurotoxicity due to oral administration or as a metabolite of induced enteric bacterial overgrowth. Establishing biomarkers of these two exposures is important for protecting children’s health by documenting the role of the imbalance in gut microbiota in the etiology of autism through the gut-brain axis. These outcomes will help efforts directed at controlling the prevalence of autism, a disorder recently related to PA neurotoxicity.

## Introduction

The investigation of the environmental contribution towards developmental neurotoxicity is fundamental to identifying the effects of environmental contaminants on humans. Exposure to environmental chemicals may contribute to the development of neurological disorders, especially in children. Animal studies may help identify the etiology of neurotoxicity due to some of these environmental chemicals. Additionally, animal studies can help understanding the protective effects of some dietary supplements against neurotoxicity. Due to the high number of reports of antibiotic exposure, hospitalization, and gastrointestinal disturbances [1-3] in many children with autism spectrum disorders (ASDs), the neurobiological effects of microbiota-produced short-chain fatty acids (SCFAs), such as propionic acid (PA) has been examined [4-7].

Finegold *et al*. (2010) [8] demonstrated that there is a significantly greater diversity of bacteria in the feces of autistic subjects compared to control subjects. The increased microflora in autistic children may contain harmful genera or species contributing to the severity of autistic symptoms [8]. Two examples are *Bacteroidetes* and *Firmicutes*. The vast majority of species in the *Bacteroidetes* phylum produce propionic acid and lipopolysaccharides (LPS), which are well-known virulence factors.

The presence and abundance of *Clostridium* in the intestines of autistic children is well documented. Finegold [9] hypothesized that the relapse of some autistic children after antibiotic treatment is due to *Clostridium* spores. The incidence of autism is related to widespread exposure to *Clostridium* spores, and the increase of multiple cases of autism within a single family is also related to contact with spores [9]. Propionate has been shown to have severe neurological effects in rats [10,11] and *Clostridia* produce propionate [12]. Studies by MacFabe *et al.* (2007) [10] have demonstrated that injecting propionate directly into specific regions of rat brains *in vivo* can cause significant behavioral problems.

MacFabe *et al.* (2007) [10] postulated that the overuse of antibiotics that has contributed to an increase in pathogenic bacteria of which *Clostridium* infections could be playing a role in the pathophysiology of autism. Additionally, clindamycin-treated hamsters are predictably susceptible to infection with pathogenic strains of *Clostridium difficile* and, as an animal model, they parallel most of the important aspects of human *C. difficile* associated disease (CDAD) [13]. A number of important observations were reported when a small amount of PA was infused into the brains of rodents [10]. Impact on socialization and behavior was also evaluated in a novel gut-mediated autism model [11]. Infusion with small amounts of PA resulted in social impairment, repetitive behavior and obsessive-compulsive behavior [14].

Concern has been expressed over the alarming increase in the rates of ASDs worldwide. Evaluation of the outcomes of oral administration of PA may aid in the understanding of these disorders. Hence, the present study was undertaken to investigate and evaluate the genotoxic effects associated with the oral administration of PA in comparison to overgrowth of intestinal microbiota induced in hamsters that received clindamycin. The study was extended to investigate the neuroprotective effects of carnosine and carnitine. Evaluation was done at the genotoxic levels through the use of tail length and tail moment to evaluate DNA damage as a marker of neurotoxicity.

## Material and methods

### Chemicals

Propionic acid, carnosine and carnitine were of analytical grade from Sigma-Aldrich. Clindamycin was purchased from Pharmacia Co., Peapack, NJ, USA.

### Animals

A total of 54 young male golden Syrian hamsters weighing approximately 80–100 g (8 weeks of age) were used in the present study. Animals were randomly allocated to one of 9 groups consisting of 6 animals each: a control group that received only phosphate buffered saline; an oral buffered PA-treated group that was given a neurotoxic dose of 250 mg/kg body weight/day for 3 days [15]; a clindamycin-treated group that received a single dose (orogastrically) of 30 mg/kg on experiment day 0; a carnosine-treated group that received a dose of 10 mg/kg body weight/day orally (daily for one week); a carnitine-treated group that received 50 mg/kg body weight/day orally (daily for one week) and; four protected groups were given the same doses of carnosine or carnitine for one week followed by PA for 3 days or a single dose of clindamycin as described above. All groups were kept at a controlled temperature (21 ± 1°C) with ad-libitum access to food and water. Quantitative stool cultures were collected and tested both aerobically and anaerobically on groups of hamsters receiving clindamycin and the untreated controls. All experiments were performed in accordance with national animal care guidelines and were pre-approved by the faculty ethics committee, King Saud University.

### Brain tissue preparation

At the end of the experiment, hamsters were anesthetized with carbon dioxide. The brain was removed from the skull and the cortex and medulla were extracted. Brain tissues of the nine groups were kept at −80°C until use.

### Single cell gel electrophoresis (comet assay)

Single cell gel electrophoresis or comet assay is a simple, sensitive and rapid method for the detection and quantification of DNA damage [16]. Slides were prepared in duplicate per group and the test was performed for at least 3 different brain (cortex and medulla) samples from each group. For the cell suspension, approximately 4 × 10^6^ cells were mixed with 80 μl of 0.7% low-melting agarose in phosphate-buffered saline (PBS) at 37°C in a microtube, and then spread over a window microscopic slide. The slides were precoated with 150 μl of 0.5% normal-melting agarose in PBS, and were specially designed for this assay. The slides were immediately placed in cold lysis buffer containing 2.5 M sodium chloride (NaCl), 100 mM EDTA sodium salt Na_2_EDTA, 10 mM Tris (pH 10), and 1% Triton X-100, at 4°C for a minimum of 1 hr. After lysis, the slides were drained and placed in a horizontal gel electrophoresis tank placed in ice, and filled with fresh cold electrophoresis buffer (300 mM sodium hydroxide (NaOH), 1 mM NaEDTA, pH 13). To allow uncoiling of DNA, the slides were kept in the high pH buffer for 20 minutes. Subsequently, electrophoresis was carried out for 20 minutes at 25 V and 300 mA. The slides were then drained and flooded slowly with 3 changes of neutralization buffer (0.4 M Tris, pH 7.5) for 5 minutes each, and then stained with 30 ml of ethidium bromide (20 mg/l) and covered with cover slips. All those steps were performed under dimmed light to prevent additional DNA damage caused by visible light. A total of 50 randomly selected cells per slide were analyzed. Imaging was performed with a fluorescence microscope (Zeiss Axiovert L410 Inc., Jena Germany), attached to a digital camera (Olympus Inc., Tokyo, Japan), and equipped with a 549 nm excitation filter, 590 nm barrier filter, and a 100-W mercury lamp. The percentage of DNA in the comet tail ("DNA damage") was automatically calculated using a ‘Toolbox’ from the IN Cell Investigator analysis package (GE Healthcare Life Sciences). Tail moment was calculated as a product of tail length multiplied by tail % damage.

## Measurement of colon microbiota

### Sample collection

Hamsters’ caecum samples were collected in sterile tubes with clindamycin- induced ileocecitis and immediately frozen at −70°C. The frozen tubes were sent to a microbiology laboratory for analysis. The process of bacterial cultivation involved the use of optimal artificial media and incubation conditions to isolate and identify the colon microbiota of an animal as rapidly and as accurately as possible.

### Isolation procedures

Stools were usually watery, with a characteristic foul odour, mucoid and soft. Gross blood in stool did occur in some cases. Five different media were employed to isolate colon microbiota. Stool samples were diluted in glycerol transport broth [17] to yield a 1/10 dilution. Five percent sheep blood agar (BAP), MacConkey agar (MAC), Muller Hinton agar (MHA), Saboraud’s Dextrose agar (SDA) (as selective medium for yeast) and modified cefoxitin cycloserine fructose agar (CCFA) (as selective medium for *C. difficile*) were used in the present study. For the quantification of C. *difficile,* a sample of 0.1 ml of serial dilution was plated on CCFA and incubated at 37°C for at least 72 h in the anaerobic jar [18]. For other aerobic microorganisms, portions of 0.1 ml of 10^-2^, 10^-3^, and 10^-4^ dilutions were plated with a glass spreader on to BAP, MAC, MHA, and SDA. The inoculated plates were incubated at 37°C for 2 days in aerobic conditions.

### Identification criteria

Colonies of *C. difficile* growing on CCFA, and blood agar were examined under long-wavelength ultraviolet light (Mineralite UVSL-25; Ultraviolet Products, Inc., San Gabriel, Calif.) for fluorescence. Whenever fluorescence, colonial morphology, or Gram stain morphology was characteristic of *C. difficile*, the isolate was identified by the criteria outlined in the Anaerobe Laboratory Manual [19]. Yellow fluorescence of the colonies of *C. difficile* on CCFA could be detected after 24 h of incubation and persisted for 5 to 6 days.

MAC or MHA and BAP media are recommended as primary plating media for most routine aerobic bacteriologic cultures. On Muller-Hinton agar plates, several bacterial species that were cultured under aerobic condition from stool samples, before and after treatment of hamsters with clindamycin, included *Streptococcus*, *Staphylococcus aureus*, *Klebsiella pneumoniae*, and *Pseudomonas aeruginosa.* There was an overgrowth of *Candida albicans* on SDA agar plates.

Identification of microorganisms of clinically significant streptococci (β hemolysis species), staphylococci, and a selected group of Gram-positive bacilli could be detected on blood agar plates after the treatment of hamsters with clindamycin. Enterobacteriaceae (lactose fermenter microorganisms) were detected on MacConkey agar media after administration of clindamycin to hamsters.

### Quantitative study of bacterial flora

The quantification of culture-based methods was based on a scale of ++++, defined as:

0 = no growth, < 10^3^ colony forming units/gram of feces

+ = Rare, less than 10^3^ colony forming units/gram of feces

++ = Few, 10^3^ - 10^4^ colony forming units/gram of feces

+++ = Moderate 10^5^ - 10^6^ colony forming units/gram of feces

++++ = Heavy > 10^6^ colony forming units/gram of feces

(Colony-forming unit (CFU) is a measure of viable bacterial or fungal numbers. Unlike direct microscopic counts where all cells, dead and living, are counted, CFU measures viable cells) For each dilution, the number of colony forming units on the plates were counted. Typically, numbers between 30 and 300 were used to estimate the culture count.

### Statistical analysis

The data were analyzed using the statistical package for social sciences (IBM Corp., New York, NY, USA). The results were expressed as mean ± S.D. All statistical comparisons between the control and PA-treated hamster groups were performed with the one-way ANOVA test complemented with Dennett test for multiple comparisons. *P* < 0.05 was statistically significant. ROC analysis was performed. Area under the curve (AUC), cutoff values, and degree of specificity and sensitivity were calculated.

## Results and discussion

The association between bacteria and inflammation is complex as either can impact the other. Bacterial overgrowth could induce different levels of activation of the various innate sensors, which can influence the gene expression pathways, level of inflammation and the results of these changes on DNA damage and chromatin alterations. Numerous studies [20-22] have verified the association between an alteration of the dominant phyla of bacteria in the gut and systemic effects in humans and animal models. Gut microbes can impact insulin resistance, inflammation, and adiposity *via* interactions with epithelial and endocrine cells [20]. The intestinal epithelial cells (IECs) have a complex and mutually beneficial relationship with the gut flora. The bacteria metabolize some nutritional components in the gut, e.g. carbohydrates; in turn, the IECs metabolize the short-chain fatty acids (e.g. propionic and acetic acid) and use them as a source of energy. The microflora in the gut is essential for processing dietary polysaccharides. The data in Table 1 indicate that clindamycin was effective in inducing intestinal overgrowth of microbiota. The most striking changes in feces of clindamycin-treated hamsters, as compared with untreated controls, included, a marked increase in total Clostridia species and *Klebsiella pneumoniae,* with a smaller increase in Group A Streptococci. The low counts of bacteria recovered from some of the specimens were likely due to several factors including dilution effects of diarrhea and the reduction in the number of viable bacteria caused by freezing and/or prolonged storage at −70°C.

**Table 1:** Estimation of clindamycin-induced changes in cecal flora of hamster

**Media used and incubation conditions**	**Control hamsters**	**Clindamycin recipients**
MHA/aerobic; 37°C/24 h	++	++++
MAC/aerobic; 37°C/24 h	0	++
BAP/aerobic; 37°C/24 h	+	++
SDA/aerobic; 25°C/48 h	+	++
CCFA/anaerobic; 37°C/72 h	0	++

**MHA:** Muller Hinton Agar.

**MAC**: MacConkey Agar.

**BAP:** 5% Sheep Blood Agar.

**SDA:** Sabouroud Dextrose agar (yeast media).

**CCFA:** modified Cefoxitin Cycloserine Fructose Agar.

The induction of pathogenic bacteria in hamsters that received clindamycin could be related to the etiology of autistic biochemical characteristics previously induced in rats that received oral propionic acid, a metabolite of some pathogenic enteric bacteria [23]. It is well documented that autistic patients show bacterial overgrowth, possibly due to excessive use of oral antibiotics (e.g. clindamycin), which can alter gut flora [24-27]. Oral antibiotics are commonly used by autistics for treating otitis media (ear infections), which occurs frequently in these patients. This observation may suggest an impaired immune system, a biochemical feature related to PA induced brain toxicity in rats [23]. Commonly used oral antibiotics eliminate almost all of the normal gut microbiota, which play an important role in the breakdown of plant polysaccharides, promoting gastrointestinal motility, maintaining water balance, producing some vitamins, and competing against pathogenic bacteria. Thus loss of normal gut flora with clindamycin could result in the overgrowth of pathogenic flora identified in Table 1. This observation is supported by Buffie *et al*’s [28] study that found a single dose of clindamycin results in the reduction of the normal diversity of the intestinal microbiota for at least 28 days and induces sustained susceptibility to *C. difficile* toxins such as propionic acid.

**Figure 1:**
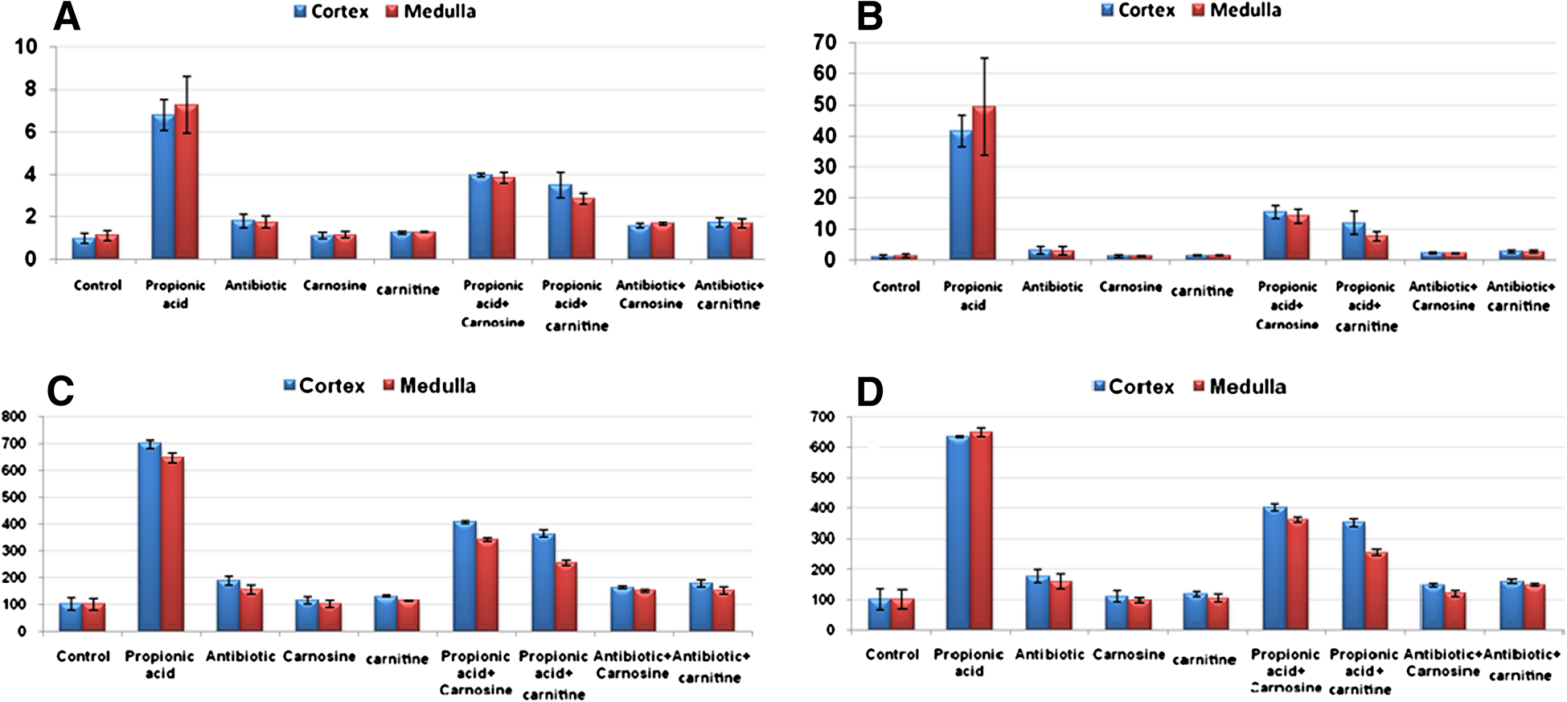
**DNA damage induced in hamster brains (cortex and medulla) by PA or clindamycin-induced bacterial overgrowth together with the protective effects of carnosine and L-carnitine.** Neurotoxic effects of PA, bacterial overgrowth and ameliorating effects of both the supplements could be seen as significant changes in tail length (μm) and tail moments (Arbitrary units) (**A** &**B** respectively) and percentage changes in both (**C** &**D**). All figures are presented as mean±SE bars in the 8 studied groups compared to a control group. It is clear that orally administered PA was more neurotoxic than induced bacterial overgrowth. Carnosine was more protective than carnitine.

**Figure 2:**
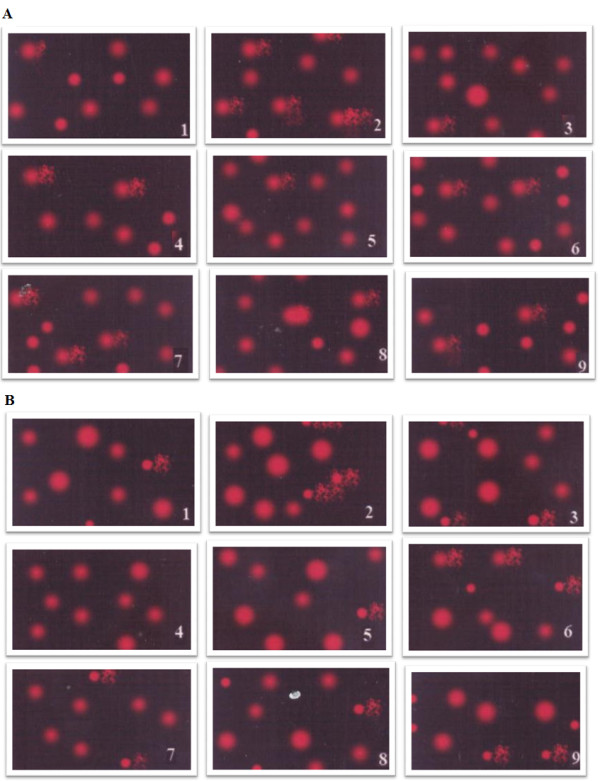
**Measure of PA or clindamycin-induced DNA damage by comet assay.** (**A**) Photograph showing comet tailing in PA and clindamycine treated hamsters together with the protective effects of carnosine and carnitine in cortex; (**B**) Photograph showing comet tailing in PA and clindamycine treated hamsters together with the protective effects of carnosine and carnitine in medulla.

Table 2, Figure 1A-D and Figure 2A & B demonstrate PA and clindamycin induced DNA damage in the cortex and medulla of treated hamsters. The evidence for DNA damage is the significant increase in the comet parameters, presented as tail length (μm) in figures A and B, tail DNA(%) and tail moment (arbitrary units). It is clear that DNA damage produced by bacterial overgrowth induced by clindamycin-treatment was not comparable to that produced by orally administered PA in the hamsters. For example, PA induced approximately a 700% increase in tail length and tail moment, whereas, bacterial overgrowth induced a 180% increase in both the parameters. Our results indicate that the measurement of the percentages of tail DNA gives additional information on the extent of DNA damage in the cortex and medulla of PA-treated hamsters. Thus, high levels of DNA double strand breaks showed increased comet tail fluorescent intensity with ethidium bromide staining. This could prove that PA not only penetrates the blood–brain barrier, but it can also induce DNA damage in brain cells. The primary mechanism of PA-induced DNA damage is not fully understood. We postulate that PA, similar to its derivative 3-nitropropionic acid (3 N-PA), could inhibit complex II of the respiratory chain leading to a rapid decline in ATP levels followed by mitochondrial DNA damage and dysfunction. ATP depletion, and mitochondrial dysfunctions are two mechanisms involved in the pathophysiology of autism [29], the postulated mechanism of PA toxicity, could confirm the role of the persisting effect of PA in the aetiology of biochemical features associated to autism as reported by El-Ansary *et al.*[23] in PA-treated rat pups. DNA strand breaks in the cortex and medulla of clindamycin-treated hamsters could be indirectly caused by PA as a metabolite of the induced pathogenic bacteria and due to the increased susceptibility of treated hamsters to *C. difficile*[28]*.* The remarkably higher effect of orally administered PA is likely related to the significant difference in the toxic dose administered.

**Table 2:** DNA damage induced in cortex and medulla of PA-treated, Clindamycin-treated, carnosine and carnitine protected groups

**Groups**		**Parameters**		
		**Tail length (μm)**	**Tail DNA %**	**Tail moment**
**Control**	Cortex	0.97 ± 0.24	0.96 ± 0.33	0.98 ± 0.59
	Medulla	1.12 ± 0.24	1.03 ± 0.32	1.21 ± 0.57
**Propionic acid**	Cortex	6.80 ± 0.74**	6.10 ± 0.20**	41.51 ± 5.09**
	Medulla	7.27 ± 1.33**	6.67 ± 0.99**	49.32 ±15.53**
**Clindamycin**	Cortex	1.81 ± 0.32*	1.71 ± 0.36*	3.16 ± 1.26*
	Medulla	1.75 ± 0.29*	1.64 ± 0.40	2.95 ± 1.25
**Carnosine**	Cortex	1.10 ± 0.14	1.05 ± 0.20	1.18 ± 0.36
	Medulla	1.15 ± 0.15	1.00 ± 0.10	1.16 ± 0.27
**Carnitine**	Cortex	1.25 ± 0.07	1.13 ± 0.09	1.42 ± 0.19
	Medulla	1.28 ± 0.03	1.07 ± 0.14	1.37 ± 0.17
**PA +Carnosine**	Cortex	3.96 ± 0.09**	3.87 ± 0.45**	15.34 ± 2.11**
	Medulla	3.83 ± 0.27**	3.69 ± 0.32**	14.19 ± 2.24**
**PA +Carnitine**	Cortex	3.50 ± 0.60**	3.37 ± 0.47**	11.96 ± 3.80*
	Medulla	2.85 ± 0.27**	2.63 ± 0.28**	7.56 ± 1.46**
**clindamycin +Carnosine**	Cortex	1.58 ± 0.10*	1.42 ± 0.09	2.25 ± 0.29*
	Medulla	1.68 ± 0.07*	1.23 ± 0.12	2.07 ± 0.12
**clindamycin+Carnitine**	Cortex	1.73 ± 0.20*	1.54 ± 0.11*	2.67 ± 0.50*
	Medulla	1.70 ± 0.21*	1.52 ± 0.06	2.60 ± 0.39*

Independent *t*-test between the control and PPA groups of Cortex and Medulla in Tailed %, Untailed %, Tail length (μm), Tail DNA % and Tail moment.

* Significant at 0.05 level.

** Significant at 0.01 level

Thus, clindamycin induced DNA damage could be attributed to the overgrowth of anaerobic bacteria, e.g. *Clostridium* that has recently been implicated as a causative factor involved in the etiology of autism [14]. This observation is supported by stool studies, which proved that certain Clostridia were important and that siblings of autistic children show intermediate Clostridia growth between healthy controls and their affected siblings [8,30].

It has been proposed that oxidative stress is related to a time-dependent shift in the antioxidant/prooxidant balance towards oxidative damage. Increased production of oxidants *in vivo* can cause damage to intracellular macromolecules such as DNA, proteins and lipids, which can in turn lead to oxidative injury. Additionally, the increased nuclear DNA damage in PA and clindamycin treated animals could be related to oxidative stress status in both groups of hamsters compared to the healthy control group. Oxidative damage by a reactive oxygen species (ROS) producing toxicity can affect DNA integrity to a different extent [31,32]. This is consistent with a recent study by El-Ansary *et al.*[23], which reported increased lipid peroxides as an index of oxidative stress, coupled with depletion of reduced glutathione and lower catalase and glutathione peroxidase activities in PA-treated rat pups.

Carnitine is a vitamin-like compound that serves as a carrier to transport long-chain fatty acids (e.g. propionic acid) into the mitochondria for beta-oxidation. In the present study, the effect of L-carnitine, a widely recognized essential nutrient, was evaluated on the status of DNA damage induced in hamsters. Table 2 also demonstrates the potency of L-carnitine in protecting against PA neurotoxicity. It ameliorates the DNA damaging effects of both treatments especially in PA-treated hamsters which had a 400% recovery for the cortex and 280% recovery for the medulla. This finding supports a recent study by Ribas *et al.*[33] who reported that propionic acidemia leads to severe metabolic complications in the neonatal period and to long-term neurological manifestations. They [33] evaluated the *in vitro* effects of PA with or without L-carnitine, on DNA damage in peripheral leukocytes, as determined by alkaline comet assay. PA induced DNA damage index (DI) was significantly higher than the control group [33]. L-carnitine significantly reduced the PA induced DNA damage, in a concentration-dependent manner [33]. Administration of L-carnitine significantly decreased the levels of lipid peroxides and improved the activities of antioxidant enzymes such as superoxide dismutase, catalase, glutathione peroxidase and glutathione reductase [34]. This group of antioxidant enzymes are affected by PA neurotoxicity and are clinically impaired in autistic patients compared to controls [23]. Additionally, a protective effect of carnitine reported in the present study is likely due to L-carnitine enhanced T-cell proliferative response and significantly reduced DNA damage, apoptosis and TNF-alpha levels in the lymphocytes of aged animals [34] and in the brain of PA-intoxicated rat pups (unpublished work).

Table 2 demonstrates the effect of carnosine in inducing 300% and 350% recovery for PA-intoxicated cortex and medulla respectively. The remarkable protective effect of carnosine reported in the present study concurs with reports. Carnosine performs critical biological functions, in particular, antioxidant properties directed at the suppression of free radical reactions [35,36]. It is well known that the imidazolium group of histidine or carnosine stabilizes adducts formed at the primary amino group and may perform a crucial function as an anti-crosslinking agent [37]. The results of many biochemical studies have suggested that carnosine harbours free radical-scavenging activity, which may partly explain its apparent homeostatic functions [38,39]. Recently, *in vitro* and *in vivo* studies have shown that carnosine can exert neuroprotective effects *via* a variety of mechanisms [40]. It has been reported that carnosine quenches 50–95% of the hydroxyl radicals generated in the Fenton reaction [41]. Therefore, it was suggested that the ability of carnosine to inhibit ferritin-mediated DNA damage was likely attributable to free radical-scavenging activity. In a more recent study, carnosine was reported to protect neurons from lipid peroxide-induced cell injury [42].

The low or non-significant effects of both carnitine and carnosine in case of clindamycin-treated samples could be attributed to the metabolic activity of the induced growth of bacteria. Bacterial metabolic activity expressed as the fermentation profile, or short-chain fatty acid profiles, may reduce the sensitivity of colonocytes to both agents [43,44].

**Figure 3:**
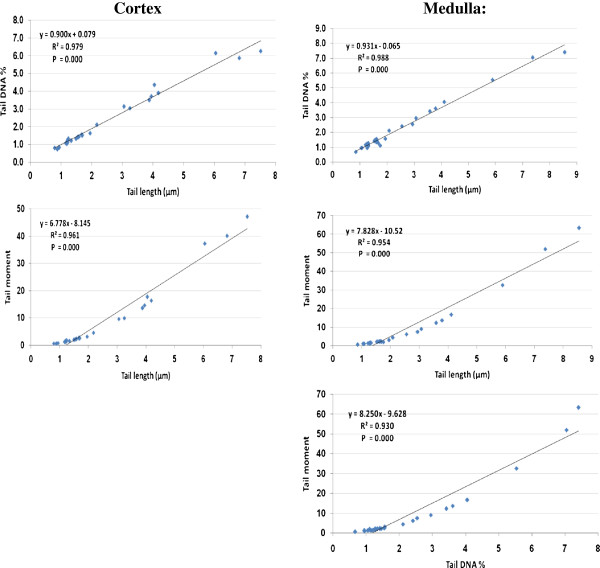
Pearson`s positive correlations with the best fit line of the three Comet assay parameters.

Table 3 shows the specificity, sensitivity of tail length and tail moment in the 8 groups studied. It is clear that the specificity and sensitivity for the parameters studied were almost 100% in PA or clindamycin-treated groups and carnosine or carnitine-protected groups. Therefore the tail length and tail moment can be used as biomarkers for PA-related neurotoxicity and for the carnosine and/or carnitine neuroprotective effects. The three measured parameters positively correlated with statistically significant *P* values (*P*<0.001, all parameters) (Table 4 & Figure 3).

**Table 3:** ROC-curve results for tail moment of brain cortex and medulla of the different studied groups showing AUC, best cut-off values, specificity and sensitivity

**Parameter**	**Group**	**Area under the curve**	**Best cutoff value**	**Sensitivity %**	**Specificity %**
**Cortex**	Propionic acid	1.000	19.465	100.0%	100.0%
	Clindamycin	1.000	1.968	100.0%	100.0%
	Carnosine	0.667	0.720	100.0%	66.7%
	Carnitine	0.667	0.950	100.0%	66.7%
	Propionic acid+Carnosine	1.000	7.640	100.0%	100.0%
	Propionic acid+Carnitine	1.000	5.642	100.0%	100.0%
	Clindamycin +Carnosine	1.000	1.822	100.0%	100.0%
	Clindamycin +Carnitine	1.000	1.931	100.0%	100.0%
**Medulla**	Propionic acid	1.000	17.147	100.0%	100.0%
	Clindamycin	1.000	1.910	100.0%	100.0%
	Carnosine	0.556	1.209	66.7%	66.7%
	Carnitine	0.444	0.882	100.0%	33.3%
	Propionic acid+Carnosine	1.000	6.954	100.0%	100.0%
	Propionic acid+Carnitine	1.000	3.905	100.0%	100.0%
	Clindamycin +Carnosine	1.000	1.794	100.0%	100.0%
	Clindamycin +Carnitine	1.000	1.963	100.0%	100.0%

**Table 4:** Pearson correlations coefficients and significance levels between DNA %, tail length and tail moment in cortex and medulla

**Parameters**		**R (Person correlation)**	**Sig.**	
Cortex	Tail length ~ Tail DNA %	0.990	0.001	P^a^
	Tail length ~ Tail moment	0.981	0.001	P^a^
	Tail moment ~ Tail DNA %	0.965	0.001	P^a^
Medulla	Tail length ~ Tail DNA %	0.994	0.001	P^a^
	Tail length ~ Tail moment	0.977	0.001	P^a^
	Tail moment ~Tail DNA %	0.965	0.001	P^a^

^**a**^_._ Positive Correlation.

Based on this study, we can suggest that the general abuse of antibiotics leads to overgrowth of Clostridia species as PA producers. The more pronounced effects of orally administered PA compared to clindamycin-treated hamsters observed in the present study could be attributed to the effect of diet which undoubtedly plays a major role in PA production as a bacterial metabolite [45].

Stated differently, key factors in the virulence of bacterial overgrowth and its mechanism leading to autism are the production of PA as an enteric bacterial fermentation product that is usually increased in the presence of a high carbohydrate diet [46]. Based on this study, a diet excluding complex carbohydrates or diets high in anti-oxidants (e.g. carnitine and/or carnosine) may subjectively give positive results in controlling the increasing prevalence of autism. This conclusion is admittedly tough to prove mechanistically and requires further study emphasizing the role of the gut-brain axis in the pathoetiology of autism [47].

## Competing interests

The authors declare that they have no competing interests.

## Authors’ contributions

AE-A: Design the work and drafted the manuscript. GS: Performed the microbiology part. AE-G: Suggested the statistical analysis used. LA-A: co-drafted the manuscript. All authors read and approved the final manuscript.
